# Testing the ‘Hybrid Susceptibility’ and ‘Phenological Sink’ Hypotheses Using the *P. balsamifera* – *P. deltoides* Hybrid Zone and Septoria Leaf Spot [*Septoria musiva*]

**DOI:** 10.1371/journal.pone.0084437

**Published:** 2013-12-27

**Authors:** Jared M. LeBoldus, Nathalie Isabel, Kevin D. Floate, Peter Blenis, Barb R. Thomas

**Affiliations:** 1 Department of Plant Pathology, North Dakota State University, Fargo, North Dakota, United States of America; 2 Laurentian Forestry Centre, Canadian Forest Service, Natural Resources Canada, Sainte-Foy, Québec, Canada; 3 Lethbridge Research Center, Agriculture and Agri-Food Canada, Lethbridge, Alberta, Canada; 4 Department of Renewable Resources, University of Alberta, Edmonton, Alberta, Canada; 5 Alberta-Pacific Forest Industries Inc., Boyle, Alberta, Canada; Portland State University, United States of America

## Abstract

Hybrid genotypes that arise between plant species frequently have increased susceptibility to arthropod pests and fungal pathogens. This pattern has been attributed to the breakdown of plant defenses (‘Hybrid susceptibility’ hypothesis) and (or) to extended periods of susceptibility attributed to plant phenologies in zones of species overlap and (or) hybridization (‘phenological sink’ hypothesis). We examined these hypotheses by assessing the susceptibility of parental and hybrid *Populus* host genotypes to a leaf spot disease caused by the fungal pathogen *Septoria musiva*. For this purpose, 214 genotypes were obtained from morphologically pure zones of *P. balsamifera* and *P. deltoides*, and from an intervening zone of overlap and hybridization on the drainage of the Red Deer River, Alberta, Canada. Genotypes were identified as *P. balsamifera*, *P. deltoides*, or hybrid using a suite of 27 species-specific SNP markers. Initially the genetic structure of the hybrid zone was characterized with 27.7% of trees classified as admixed individuals. To test the hybrid susceptibility hypothesis, a subset of 52 genotypes was inoculated with four isolates of *S. musiva*. Levels of susceptibility were *P. balsamifera* > F_1_ hybrid > *P. deltoides*. A further 53 genotypes were grown in a common garden to assess the effect of genotype on variation in leaf phenology. Leaf phenology was more variable within the category of hybrid genotypes than within categories of either parental species. Leaf phenology was also more variable for the category of trees originating in the hybrid (*P. balsamifera* – *P. deltoides* [hybrid and parental genotypes combined]) zone than in adjacent pure zones of the parental species. The results from the inoculation experiment support the hybrid intermediacy hypothesis. The results from the common garden experiment support the ‘phenological sink’ hypothesis. These findings have greatly increased our understanding of the epidemiology and ecology of fungal pathogens in plant hybrid zones.

## Introduction

Natural hybrid zones frequently have been observed to support greater abundances of insects and pathogens than the parental host species [1]. Within these zones, hybrid genotypes may be comprised of F_1_ intermediates and a range of complex backcrosses [2], [3] with different levels of susceptibility both within and among hybrid categories. For simplicity, however, these different classes of genotypes have been combined into one category (hybrid) to compare the overall effect of hybridization on host susceptibility. Reviews of the literature in this fashion reveal a range of responses [1], [2], [3] that form six testable hypotheses; i.e., four genetic models [2], [3] and two ecological models [3], [4], [5]. The genetic models do not apply to the inheritance of specific genes or groups of genes, but rather to overall changes in resistance and susceptibility. They are defined as follows: (i) hybrid susceptibility: hybrids are more susceptible than either parental species; (ii) hybrid resistance: hybrids are more resistant than either parental species; (iii) ‘additive hypothesis’: hybrids are intermediate in resistance as compared to both parental species; and (iv) the ‘dominance hypothesis’: hybrids have the same resistance or susceptibility as one of the parental species [2].

Superimposed on these genetic models are two ecological models that attempt to explain parasite population dynamics. The first of these is the ‘hybrids as sinks’ hypothesis. It proposes that susceptible hybrids not only have greater parasite loads, but that they may draw parasites away from the parental species [4]. The second of these is the ‘phenological sink’ hypothesis which suggests that zones of overlap and hybridization between plant species are more variable in phenology than zones of non-overlap, with associated implications for the enhanced susceptibility of mixed stands to arthropods and pathogens [5]. McIntire and Waterway [6] observed this phenomenon in a smut – hybrid *Carex* pathosystem in northern Canada. In that system, *Carex* hybrids had a longer period of susceptibility to a smut fungus than did pure species. Although the authors observed an increased period of susceptibility corresponding with greater levels of disease on hybrids compared to pure species, the ‘phenological sink’ hypothesis was not formally tested. The ‘hybrids as sinks’ hypothesis is unlikely to be relevant for plant pathosystems because pathogens do not actively seek out their hosts as do insects. However, the ‘phenological sink’ hypothesis may play an important role in disease epidemiology similar to that observed by McIntire and Waterway [6].

Natural hybridization is common in the genus *Populus* [7]. Hybrid populations are regularly found wherever species of *Populus* from the Sections *Aigeiros* and *Tacamahaca* are sympatric [8], [9]. The small number of studies examining resistance to pathogens in *Populus* hybrids has focused on controlled crosses and specific pedigrees rather than exploring susceptibility in natural hybrid populations. For example, three studies examining the response of a three generation pedigree of *Populus trichocarpa* Torr. and Gray. x *Populus deltoides* Rybd. (*P. x generosa* Henry) to natural infection in the field reported a variety of responses depending on the pathogen being observed. In the case of *Septoria populicola* Peck, a pathogen causing necrotic leaf lesions [10], and *Melampsora occidentalis* Jacks., a leaf rust, the pattern was dominance of resistance [11]. In contrast, for Septoria canker (*Septoria musiva* Peck), susceptibility appeared dominant in the F_1_ generation [12]. To our knowledge, no study has examined the response of a range of tree genotypes collected from a natural hybrid zone of *Populus* species to any pathogen nor has the ‘phenological sink’ hypothesis been tested.

**Figure 1:**
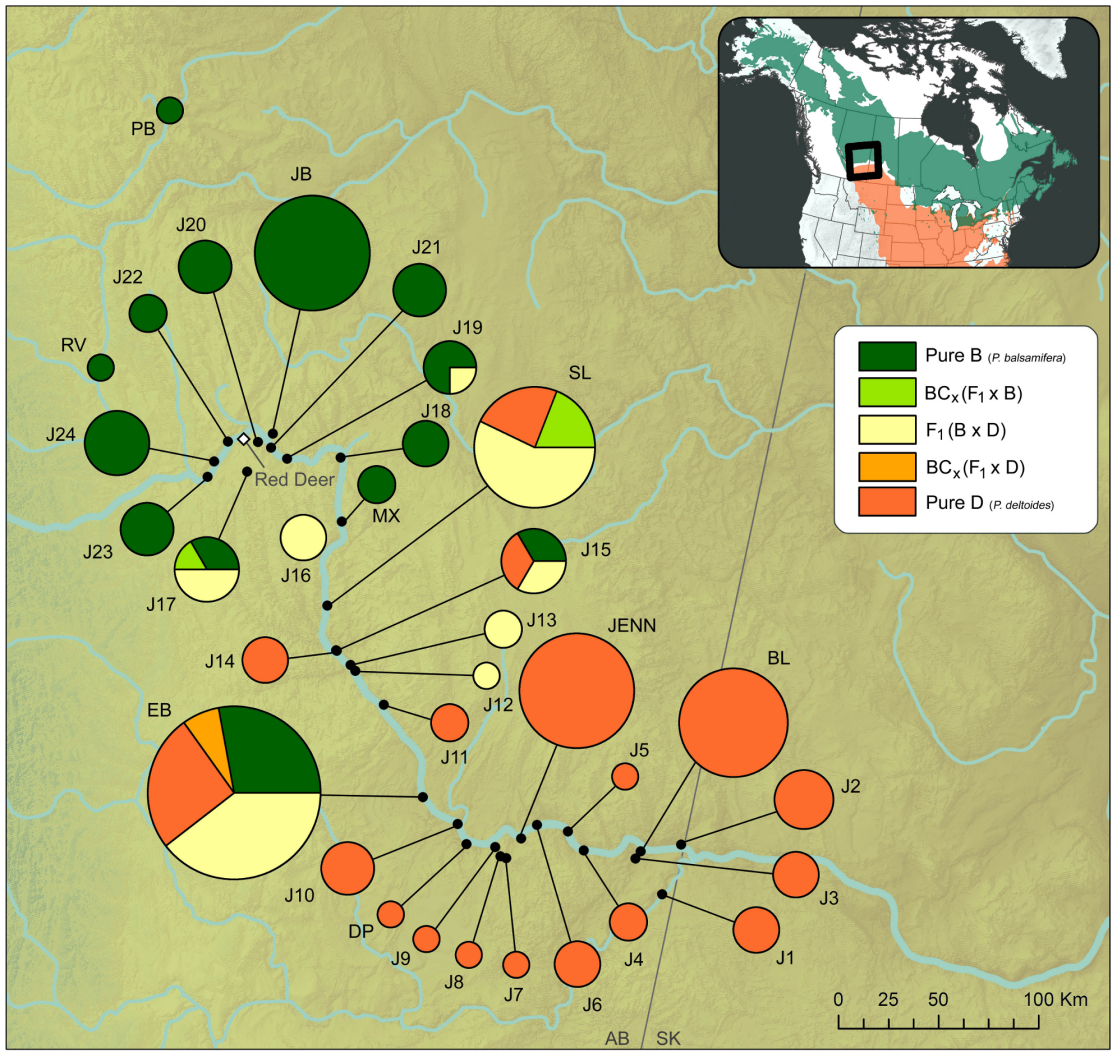
Map of sampling locations in the Red Deer River drainage of southern Alberta, Canada. Studied populations of *Populus* are indicated by black dots. The pie charts indicate the relative proportion of individuals in each genotype class (Pure *P. balsamifera* (B), Pure *P. deltoides* (D), F_1_ (BxD), backcross B, and backcross D). The size of each pie chart reflects the number of individuals genotyped in each population. The inset depicts the ranges of *P. balsamifera* and *P. deltoides* in North America.

To assess the effect of hybridization in *Populus* to pathogen susceptibility, a natural hybrid zone located in the drainage of the Red Deer River of south central Alberta was selected as a study system. This drainage includes an upstream zone of morphologically pure *Populus balsamifera* L. (balsam poplar), a downstream zone of morphologically pure *Populus deltoides* Bartr. ex. Marsh. var. *occidentalis* Rybd. (plains cottonwoods), and an intervening zone of overlap and hybridization (Figure 1) for which patterns of leaf morphology identify unidirectional introgression towards the *P. balsamifera* parent [8]. Based on molecular markers, a similar trend was observed in eastern Canada along a bi-specific zone of contact between *P. balsamifera* and *P. deltoides* [9], [13].

The hybrids between *P. balsamifera* and *P. deltoides* are widely recognized as hosts of the fungal pathogen *S. musiva* (teleomorph  =  *Mycosphaerella populorum* Thompson) [14], [15], [16], [17]. This pathogen causes both leaf spot and stem cankers depending upon the host genotype. Both *P. balsamifera* and *P. deltoides* leaves can be infected by this pathogen [17]. However, *P. balsamifera* is more susceptible to leaf spot caused by *S. musiva* than is *P. deltoides* [17]. In the case of severe infection this disease may cause premature defoliation [18].

Using the drainage of the Red Deer River as a model system, we examined the susceptibility of the two pure parental species, *P. balsamifera* and *P. deltoides*, and their hybrids to different isolates of *S. musiva*. Specifically, we: (i) selected a haphazard sample of trees from the two pure zones and from the hybrid zone; (ii) used genetic markers to classify these trees as either pure *P. balsamifera* (B), pure *P. deltoides* (D), or hybrids (F_1_); (iii) inoculated a subset of these genotypes (n = 52) with four isolates of *S. musiva* to evaluate their resistance to Septoria leaf spot; and (iv) propagated a second subset of genotypes (n = 53) in a common garden to characterize variation in leaf phenology. The overall aim of this work was to characterize the genetic structure and pattern of hybridization of *Populus* spp. in the Red Deer River drainage in order to evaluate the ‘hybrid susceptibility’ and ‘phenological sink’ hypotheses on the epidemiology of a common leaf spot disease.

## Materials and Methods

### Host collection and propagation

Dormant cuttings were obtained in February 2008 from 214 trees at 33 locations in the drainage of the Red Deer River (Figure 1 and Table S1). No samples were collected in national parks or other areas that required specific permissions. Samples were collected from trees growing in flood plains. In Canada, flood plains are owned by the Crown. Public access to Crown lands is enshrined in Alberta’s Public Lands Act: (http://www.qp.alberta.ca/1266.cfm?page=p40.cfm&leg_type=Acts&isbncln=9780779743100). *P. balsamifera* (http://plants.usda.gov/java/profile?symbol=poba2) and *P. deltoides* (http://plants.usda.gov/java/profile?symbol=pode3) are common throughout North America. Neither species nor their hybrids are protected under federal or provincial laws. Source trees were collected in zones of pure *P. balsamifera* (B), pure *P. deltoides* (D) and hybridization (*P. balsamifera* – *P. deltoides*; hereafter designated B – D) as defined by Floate [8] based on leaf morphology. At each location, trees were (i) at least 30 m apart to ensure that collections were from different clones; and (ii) had crown architecture, bud morphology, and bark characteristics similar to *P. deltoides*, *P. balsamifera*, or *P. balsamifera* x *P. deltoides* (F_1_ hybrid). Cuttings (10-cm in length, 20/tree) were collected from the previous year’s growth, placed in plastic bags, labeled, and stored between 0°C to −3°C, for approximately three days. After returning to the University of Alberta, cuttings were soaked in distilled water for 48 hours at 4°C [19], and planted into 12×5×5 cm (depth × width × length) Rootrainers (Spencer-Lemaire® Rootrainers; Spencer-Lemaire Industries, Edmonton, AB) containing Metromix® 290 growing medium (Terra-Lite 2000 series; WR Grace and Company, Ajax, ON) with only the top-most bud remaining exposed above the growing medium surface.

Rooted cuttings were maintained in a greenhouse at 20/15°C (day/night) with an 18 h photoperiod supplemented with artificial lights and fertilized once a week with a 500 ppm solution of 15-30-15 fertilizer (Plant Products Company Ltd., Brampton, ON). The irradiance varied with cloud cover, but was, on average, approximately 450 µmol photons m^-2^s^−1^ PAR (photosynthetically active radiation) at the Rootrainer level. The ten tallest trees (30–50 cm) from each clone were transplanted from the Rootrainers into 18×15 cm (height × diameter) plastic pots (Listo Products Ltd., Vancouver, BC) containing Metromix® 290 growing media amended with 2.73 g L^−1^ Nutricote® 100 day slow release 13-13-13 fertilizer (6.5% NO_3_-N, 6.5% NH_4_-N, 13% P_2_O_5_, 13% K_2_0, 1.2% Mg, 0.02% B, 0.05% Cu, 0.2% chelated Fe, 0.06% Mn, 0.02% Mo, 1.3% EDTA; Plant Products Company Ltd., Brampton, ON). After transplanting, the trees were placed in cold frames outside on June 1^st^ 2008, and fertilized bi-weekly with a 500 ppm solution of 20-20-20 fertilizer (Plant Products Company Ltd., Brampton, ON).

### Molecular fingerprinting of host genotypes

#### DNA extraction and genotyping

For the SNP (Single Nucleotide Polymorphism) analysis, DNA was extracted from dried buds or leaves using a Nucleospin® 96 Plant II kit (Macherey-Nagel, Bethlehem PA) and a vacuum manifold according to the manufacturer's protocols with the following two modifications: (i) cells were lysed using buffers PL2 and PL3 for 2 h at 65°C and; (ii) DNA was eluted with an in-house Tris-Cl 0.01X pH 8.0 buffer. This procedure was adapted from that of [20]. All 214 individuals were genotyped using two different SNP arrays: one with 27 diagnostic SNPs (12 SNPs specific to *P. balsamifera*, and 15 to *P. deltoides*) derived from previous studies [13], [20], [21] and a second one, including 37 SNPs that vary at the intraspecific level for *P. balsamifera* (N. Isabel, unpublished). The latter was used only to discard duplicate genotypes. Genotyping was conducted at McGill University in the Génome Québec Innovation Center (MUGQIC) using Sequenom iPlex Gold technology and their internal protocols.

#### Admixture analysis

When duplicate genotypes were observed [21], [22], the duplicates were deleted and only a single individual was used in subsequent analyses. The 198 resulting genotypes were analyzed using Structure version 2.3.3 [23]. The 198 genotyped trees were included, as well as 60 pure individuals (30 *P. balsamifera* and 30 *P. deltoides*), as learning populations. These individuals were considered as representative of a given species (and its natural range) based on their leaf morphology and genotypes. They are listed in Table S2. The admixture modeling was conducted with the following parameters: (i) allele frequencies were assumed to be correlated; (ii) an individual alpha (Dirichlet parameter for degree of admixture) for each population with a gamma prior was used; (iii) different values of F*_st_* were assumed for different subpopulations; and (iv) a default value of 1 was used for 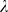
(the allele frequency prior). The Markov Chain Monte Carlo procedure was run for an initial burn-in of 100 000, followed by 500 000 replicates. Analysis of population admixture at K = 1 to 6 were conducted using the parameters described above (K is defined as the number of populations [23]). Ten independent runs of Structure were conducted at each of these values of K and the procedure developed by Evanno *et al.* [24] was used as a guide to determine the most appropriate value of K. Once an appropriate value of K was chosen, credible intervals for the admixture estimates were calculated with the ANCESTDIST function.

#### Genotype class assignment

Using the results from the admixture analysis of the 27 SNP diagnostic panel (the first SNP array), and the credible intervals calculated by Structure, individuals were assigned to one of five genotype classes, pure B, pure D, F_1_ hybrids, or BC_x_ (F_1_ × B, F_1_ × D) (Table 1). Individuals with a high probability of membership (credible interval overlapping 0.95) in either the *P. balsamifera* or *P. deltoides* genotype classes were assigned to that class. Individuals with credible intervals that overlapped 0.5 were classified as F_1_ hybrids. Individuals with credible intervals that did not overlap 0.5 or 0.95 were classified as backcross hybrids; i.e., the progeny of F_1_ hybrids and either *P. balsamifera* (BC_x_: F_1_ × B) or *P. deltoides* (BC_x_: F_1_ × D). The direction of the backcross was determined by the species with the highest associated posterior probability. For example, if Structure indicated that individual A had a 0.25 posterior probability of being *P. balsamifera* and 0.75 posterior probability of being *P. deltoides*, then that individual was considered a backcross hybrid introgressed towards *P. deltoides*.

**Table 1:** Total number of individuals per genotype class.

Genotype class	Number
*P. balsamifera* (B)	65
*P. deltoides* (D)	84
*P. deltoides x P. balsamifera* (F_1_)	41^1,2^
BC_x_ 3 (F_1_ x B)	5^4^
BC_x_ (F_1_ x D)	3^4^
Total	205

^1^ Three of these F_1_ hybrids have *P. balsamifera* as the maternal parent.

^2^ Two of the F1 hybrids have unknown maternal and paternal genotypes.

^3^ BC_x_ = Backcross to a pure species.

^4^ Maternal and paternal genotypes are unknown.

#### Chloroplast haplotyping

In order to ascertain their maternal species lineage (B or D), all individuals considered hybrid (F_1_ or BC_x_) were analysed using five polymorphisms in the chloroplast *trnL* intron [20].

### *Septoria musiva* inoculation trial

#### Pathogen collection, propagation, and inoculation

Four isolates were obtained by collecting Septoria cankers from dormant *P. balsamifera* trees at the Alberta-Pacific Forest Industries Inc. (Al-Pac) mill site in northern Alberta (54°53′ N, 112°51′ W, 575 m elevation). Cankers were first soaked in a 5% NaClO solution for 120 secs and then rinsed with sterile distilled water. The bark was then removed from the canker, exposing the margin between healthy and necrotic tissue. From this margin, a 5-mm sliver of necrotic tissue was removed and placed on a Petri dish with *Septoria musiva* media (SMM; 180 ml V8 juice, Campbell Soup Company, Camden, NJ, USA; 2 g Calcium carbonate; 20 g agar, Difco, Franklin Lakes, NJ, USA; 820 ml de-ionized water; chloramphenicol 300 mg L^−1^, Sigma-Aldrich, St-Louis, MO, USA; and streptomycin sulphate 25 mg L^−1^, Sigma-Aldrich, St-Louis, MO, USA) [25]. These plates were then sealed with Parafilm® and placed at approximately 20°C under continuous light with Gro-Lux© wide spectrum fluorescent bulbs (Sylvania; Osram Gmbh, Munich, Germany). After 7 days, sporulating fungal colonies were transferred to a second SMM plate. Once a single pure fungal colony arose, it was transferred to K-V8 growth media and allowed to grow until sporulation occurred. Species identity was confirmed using both conidial morphology [26] and species-specific markers from the internal transcribed spacer (ITS) region of the mitochondrial small subunit developed by Feau *et al.* [27]. All cultures were stored at −90°C in cryogenic vials (Nalgene® labware, Rochester, NY USA) containing 1 ml of a 50% glycerol solution.

Four cryogenic vials, containing four different isolates, were removed from cold storage to conduct the inoculation experiment. The contents from each vial were brought to room temperature, poured onto separate Petri dishes containing K-V8 growth media, and sealed with Parafilm. Subsequently, these Petri dishes were placed on the light bench described above for five days. From each of the four Petri dishes, masses (5 mm diameter) of sporulating mycelium were excised and transferred to new Petri dishes. Four masses of mycelium were placed on each dish. A total of 20 Petri dishes per isolate were created, sealed with Parafilm and placed on the light bench described above for 14 days. The inoculum suspension was created by dislodging the conidia in sterile distilled water from the surface of each Petri dish. The concentration of each suspension was adjusted to 1×10^6^ conidia L^−1^ and combined into a single mixed suspension used for inoculation [28].

Due to constrained greenhouse space, a subset of 52 individuals (13 pure B, 25 pure D, 14 F_1_ hybrids) was used in the inoculation experiment. Backcross hybrids were excluded. Five ramets from each individual, propagated and potted as described above, were arranged in a randomized complete block design with five blocks, one tree per clone per block. Each block was in a separate cold frame with genotypes randomly arranged within.

Trees were inoculated 70 days after transplanting from Rootrainers into pots. Inoculations occurred over five consecutive days, with a different block inoculated each day. The top six leaves of each tree were sprayed with inoculum using standard 500 ml spray bottles until water dripped from the leaves. Following inoculation, trees were placed in black plastic bags, two trees per bag with four wet paper towels, to maintain relative humidity [28]. The bags were sealed for 48 hours and placed in a dark room at 26.5°C. Four of the five blocks were sprayed with the spore suspension. The fifth block was a control inoculated with sterile distilled water. Following the 48 hour incubation period, each block was returned to its cold frame for three weeks. The six inoculated leaves from each tree were removed and pressed in a plant press until dry. Pressed leaves were then scanned using an HP digital scanner (© Hewlett-Packard Development Company, L.P. Palo Alto, CA USA) at a resolution of 199 dpi (dots per inch). ASSESS 2.0 [29] was used to calculate the proportion infected area (PIA) of each leaf.

#### Statistical analysis

The PIA was averaged over the six leaves for each tree and statistical analyses were performed using the SAS Mixed procedure [30] with significance assessed at α = 0.05. The statistical model used in the analysis was:
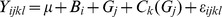



i = 1−4; j = 1−3; k = 1−52

Where:


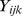
  =  PIA for the i^th^ block in which the k^th^ individual is nested within the j^th^ genotype class;


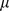
 =  the overall mean;


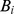
  =  the random effect of the i^th^ block;


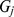
  =  the fixed effect of the j^th^ genotype class;


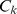
  =  the random effect of the k^th^ clone nested within the j^th^ genotype class;


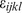
  =  the residual error.

The likelihood ratio chi-squared test was used to determine if including block in the model improved goodness of fit relative to a simpler model that did not. Pairwise t-tests of PAI values among the different genotype classes were performed using the lsmeans statement and pdiff option in SAS PROC MIXED (30). Bonferroni corrections were applied to maintain an experiment-wise error of 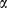 = 0.05. Degrees of freedom were estimated using the Kenward-Rogers procedure (30).To determine if the variation among clones (nested within genotype classes) differed for the three genotype classes, the likelihood ratio chi-squared test was used to evaluate if a model including heterogenous variances for genotype improved goodness of fit relative to a simpler model that assumed that the variation among clones within genotype class was equal for all genotype classes.

### Common garden phenology study

#### Common garden experiment

An additional five ramets from each of 53 individuals (13 pure B, 25 pure D, 15 F_1_ hybrids) were planted at the beginning of June 2008 in a common garden experiment at the University of Alberta Research Station located in Edmonton, AB (53°32′ N, 113°29′ W). Backcross hybrids were excluded. The experimental design was a randomized complete block design with five blocks. Each block contained 100 randomly arranged trees (one of each of the 53 genotypes and 47 fill trees). Blocks were made up of 10 rows of 10 trees with an inter-tree and inter-row distance of 1.5 m. A single border row of excess trees surrounded all five blocks. Trees were irrigated as needed for the remainder of the first growing season. Throughout the course of the 2-year trial, competing vegetation was removed on a biweekly basis by rototilling between rows and hand weeding around the base of each tree as required.

Trees were monitored throughout 2009 to assess bud phenology (time to bud burst and bud set) and to determine growing season length. Beginning on May 7^th^, trees were examined daily, with leaf emergence (bud burst) recorded as the date when the first leaf was completely unrolled (Figure S1). Beginning on July 1^st^, apical shoots of each tree were monitored weekly for signs of bud formation. When bud formation appeared imminent, monitoring was increased to every other day until buds were formed (Figure S2). Dates of first leaf emergence and bud set across all five blocks were converted to a Julian date to calculate growing season length (i.e., date of bud set minus date of first leaf emergence).

A two-step process was used to determine if there were differences in variability among clones (nested within genotype classes) for the three different genotype classes. This was done for each of the phenological variables: 1) bud burst date, 2) bud set date and 3) length of growing season. First, the modeling process described above was used to calculate average values (estimated Best Linear Unbiased Predictions) of the three different variables for each clone. Subsequently, paired F-tests were used to determine if the variability among clones differed among the genotype classes. The same process was used to determine if variances among clones differed among collection zones. (For analysis of both genotype class and collection zone effects, α was set at 0.1 because the larger of the two tested variances was always used as the numerator).

## Results

Data can be accessed at: http://www.datadryad.org/.

### Molecular fingerprinting of host genotypes

#### Admixture analysis

**Figure 2:**
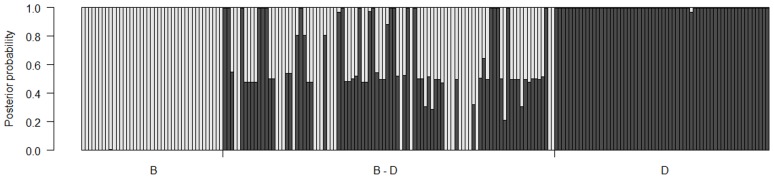
Genomic composition of sampled trees. The 198 trees sampled were genotyped using 22 interspecific SNPs, presented by collection location in the drainage of the Red Deer River, Alberta, Canada.

The genotyping indicated the presence of several genotypes that were subsequently removed from further analyses (214 sampled trees – 11 duplicate genotypes – 5 missing genotypes = 198 individuals in the final analysis). Of the 11 duplicate genotypes, 18.8% (2/11) were *P. balsamifera* and 81.2% (9/11) were F_1_ hybrids. Using the analysis described by Evanno *et al.* [24], the 27 interspecific SNPs indicated that K = 2 was the most appropriate number of clusters for the remaining 198 sampled trees, which corresponds to the number of species involved in this study. As K values increased beyond 2, the admixture estimates increased evenly across all individuals to indicate values were too high. Once the appropriate number of clusters (K = 2) had been determined, each genotype was assigned to one of five genotype classes using the credible intervals estimated by STRUCTURE. Thus, 65 individual trees were identified as *P. balsamifera*, 84 were identified as *P. deltoides*, and the remaining individuals (49) were identified as hybrids (F_1_ or BC_x_) (Table 1) (Figure 2). The percentage of hybrid individuals sampled in this study along the Red Deer River was estimated as 27.7% (55 hybrids/198 individuals), with hybrid individuals found in 8 of the 33 sampled populations (Table S1).

Of the admixed individuals, 41 were identified as F_1_ hybrids (extreme upper and lower bounds of their posterior probabilities were 0.781 and 0.322 respectively), five were identified as backcross hybrids introgressed to *P. balsamifera*, (F_1_ × B) and three were identified as backcross hybrids introgressed to *P. deltoides* (F_1_ × D; Table 1).

#### Chloroplast haplotyping

Of the 49 admixed individuals, the chloroplast *trnL* intron identified 73.4% (36/49) and 6.0% (3/49) having *P. deltoides* and *P. balsamifera* as the maternal parent, respectively. The maternal lineage of the remaining trees (20.4%: 10/49) could not be determined.

### *Septoria musiva* inoculation trial

#### Disease resistance phenotyping

**Figure 3:**
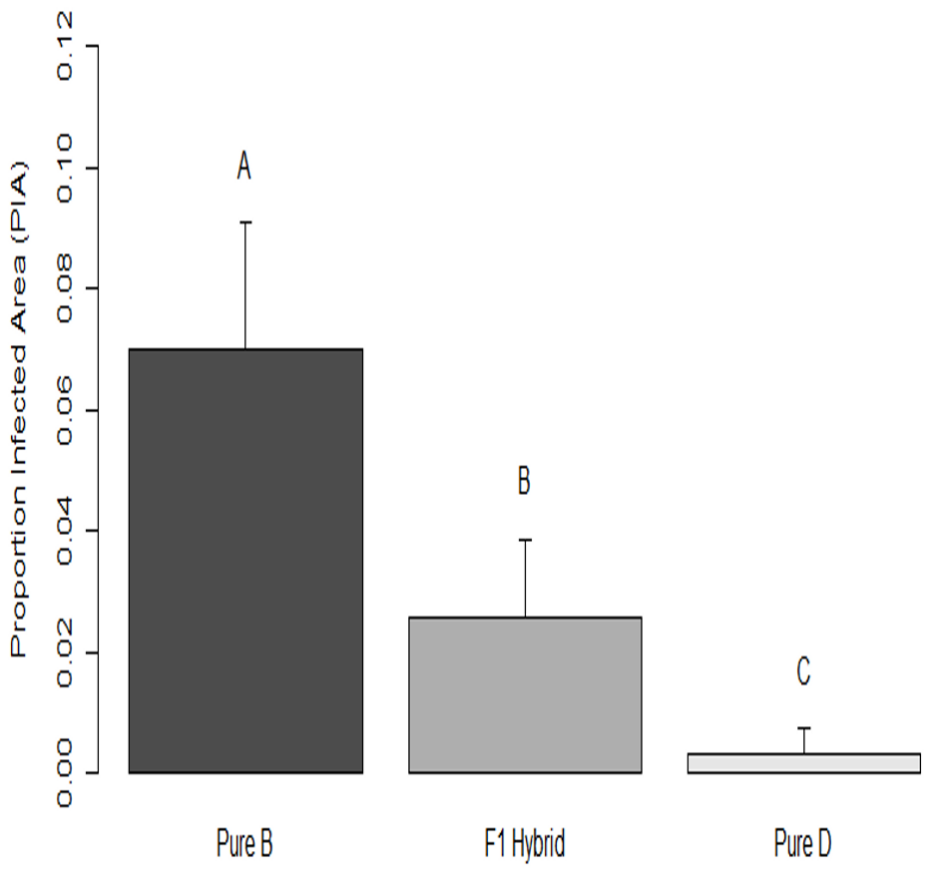
Disease severity measured as the proportion infected area and 95% confidence interval of the mean. The three genotype classes (Pure B, F_1_ Hybrid, and Pure D) are represented with bars. Bars with different letters above them are significantly different (P<0.10) from each other.

The 52 inoculated clones were assigned to one of three genotype classes (B = 13, F_1_ = 14, D = 25). The average disease severities among the three classes as determined by PIA were significantly different (Figure 3). Pure B was the most susceptible (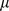 = 0.070); pure D was the most resistant (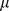 = 0.003); and F_1_ individuals were intermediate (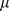 = 0.026). In particular, B was different from both the F_1_ hybrids (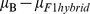 = 0.044; df = 20; P = 0.004) and D (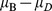 = 0.067; df = 12; P<0.001) and the F_1_ hybrids were different from D (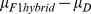 = 0.023; df = 15; P = 0.005). The likelihood ratio chi-squared test indicated that a model assuming different variances among clones within genotype classes fit the data better than a simpler model that assumed equality of clonal variances among genotype classes (P<0.001). The PIA of all control trees was 0, so controls were not used in any analyses.

### Common garden phenology study

#### Phenology phenotyping

Clonal variance in first leaf emergence differed between B and F_1_ and between D and F_1_ (both P<0.001), but not between zones B and D (P = 0.827) (Table 2). Clonal variability in bud set was not significantly different for any of the pairings (P>0.100) (Table 2). There were significant differences in clonal variance of growing season length between B and F_1_ (P = 0.092) and between D and F_1_ (P = 0.002), but not between zones B and D (P = 0.295) (Table 2).

**Table 2:** Variance among clones in different genotype classes (Pure B, F_1_ Hybrid, Pure D) and zones of collection (B, B–D, D) for the three phenological variables.

	Genotype class 1			Collection zone 2		
Phenological variable	Pure B	F_1_ Hybrid	Pure D	B	B - D	D
Bud break	1.3a	43.8b	1.5a	1.3a	44.2b	1.6a
Bud set	15.9ns^3^	9.7ns	8.9ns	14.2ns	11.8ns	8.7ns
Growing season length	18.7ab	50.5a	11.4b	17.1ab	43.2a	11.5b

Numbers with different letters are significantly different (P<0.10).

^1^ Pure B  =  P. balsamifera, Pure D  =  P. deltoides, or F_1_ Hybrid  =  P. balsamifera x P. deltoides.

^2^ B  =  *P. balsamifera* zone, D  =  *P. deltoides* zone, and B – D  =  zone of *P. balsamifera* – *P. deltoides* hybridization.

^3^ ns  =  no significant differences.

Clonal variance in first leaf emergence differed between zones B and B–D and between D and B–D (both P<0.001), but not between zones B and D (P = 0.833) (Table 2). For bud set, there were no significant differences among any of the pairings (Table 2). Finally, there was a difference in the variance in growing season length between B and B–D zones (P = 0.004) and no significant difference between B vs. B–D zones (P = 0.138) and the B and D zones (P = 0.423) (Table 2).

## Discussion

### Genotype assignment and pattern of introgression

The pattern of hybridization observed in this study is broadly consistent with that reported by Floate [8]. The SNPs identified a zone dominated by *P. balsamifera* at higher elevation, a zone dominated by *P. deltoides* at lower elevation, and an approximate 115 km zone of overlap and hybridization (Figure 1). However, hybrid individuals were detected as far northwest as the city of Red Deer. This is a much wider range than that originally reported by Floate [8]. One possible explanation for this discrepancy is the increased sampling intensity (198 individuals) in this study compared to that of Floate's (102 individuals) [8], which would increase the probability of detecting rare genotypes. A second possibility is the greater likelihood of detecting hybrids with molecular markers than with leaf morphology. However, the majority of hybrids were F_1_'s for which detection with leaf morphology is fairly reliable. A third possibility is that hybrids detected beyond the borders of the hybrid zone reported by Floate [8] have been transplanted from elsewhere as is common in rural Canada [20], or are the progeny of such hybrids. For example, collection sites J17 and J19 are in close proximity to campgrounds or shelterbelts (Figure 1).

The majority of the F_1_ hybrids (76%) have *P. deltoides* as the maternal parent and only 5.5% of hybrids have *P. balsamifera* as the maternal parent. Asymmetrical hybridization towards *P. deltoides* in F_1_ hybrids was attributed to multiple causes involving prezygotic (such as phenology of flowering) barriers, postzygotic barriers (such as conflicting developmental schedules between maternal tissue and embryo) and/or their interactions in Eastern Canada (Roe *et al.* submitted).

The molecular markers also identified eight advanced generation hybrids (BC_x_) (Table 1); i.e., five backcrosses to *P. balsamifera* and three backcrosses to *P. deltoides*. The direction of the backcross appears to be related to geographic location. Hybrids backcrossed to *P. deltoides* were found in close proximity to the morphological pure zone of *P. deltoides*, whereas hybrids backcrossed to *P. balsamifera* were found in proximity to the morphological pure zone of *P. balsamifera*. This may reflect the relative size of the local pollen clouds in these regions (Figure 1). The low frequency of advanced generation hybridization is consistent with reports for these same species in Quebec [13].

### The effect of hybridization on disease resistance

The results from the inoculation experiment (Figure 3) support the hybrid intermediacy hypothesis [2], [3]. Leaf spot severity in F_1_ hybrids was intermediate to that of the parental species (Figure 3). Hybrid intermediacy is said to be a result of additive resistance characters inherited from the two parents [3]. In the Pacific Northwest a closely related species, *Septoria populicola* Peck., exhibits a similar pattern of resistance to leaf spot infection on the *P. deltoides – P. trichocarpa* – *P. trichocarpa* x *P. deltoides* hybrid complex [10]. In this complex, *P. deltoides* is resistant, *P. trichocarpa* is susceptible and the hybrid *P. trichocarpa* x *P. deltoides* is intermediate [10]. That study identified two dominant QTL's explaining 70% of the genetic variance in resistance [10]. These QTL's were inherited from the *P. deltoides* parent [10]. Given the phenotypes observed in the inoculation experiment described above and the similarity of the two pathogens, resistance may be governed by a similar genetic mechanism in this system; whereby hybridization of the resistant *P. deltoides* and susceptible *P. balsamifera* results in intermediate resistance in the *P. deltoides* x *P. balsamifera* genotypes.

### The effect of hybridization on phenology

The ‘phenological sink’ hypothesis proposes that in hybrid zones there is greater variability in the date of first leaf emergence at the stand level, consisting of a mix of different genotype classes, compared to stands of pure species. This results in a wider range of tissue susceptibilities at the time of spore release in mixed versus pure species stands [8]. Table 2 indicates that in terms of the date of first leaf emergence hybrid genotypes (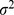 = 43.8) are more variable than either *P. balsamifera* (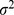 = 1.3) or *P. deltoides* (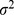 = 1.5), which supports the ‘phenological sink’ hypothesis. This result may become important if the timing of inoculum production is considered. Previous studies have indicated that peak ascospore release occurs at the beginning of the growing season and is dependent upon precipitation [31]. Although ascospore release and bud swell are roughly synchronous, there is significant year-to-year variation [31]. The greater variability in the date of first leaf emergence would increase the probability that susceptible leaves will be present at the time of peak ascospore release, maximizing the levels of initial infection.

In the common garden study, variation in growing season length and the date of first leaf emergence, for comparisons of genotypes grouped by zones, showed greater variability among genotypes from the B–D zone than that exhibited among genotypes from B or D zones (Table 2). One possible explanation of this phenomenon is the greater latitudinal range of collection locations in the B*–*D zone compared to the B and D zones. This seems unlikely, given that the entire sampled region is only 150 km in length. It is more likely that the greater variability in the hybrid zone is due to inherent variability in the hybrids compared to pure species. The lack of a significant regression between the date of first leaf emergence and collection location support this hypothesis (data not shown).

In a simplified sense, the epidemiology of any plant pathosystem is dependent on changes in susceptibility, inoculum production, and pathogen dispersal over time. There are many epidemiological parameters that were not measured in this study, which need to be considered even in a simplified framework. Inoculum production is one such parameter. No information is available on the interaction between any environmental factor and the amount of inoculum being produced. Nor is there information on how leaf age or development (ontogeny) affects inoculum production. Both environment and ontogeny have well described effects in other plant pathosystems [32], [33]. Further, other phenological characteristics of the host plants should be considered. Total leaf area may affect disease incidence and severity by increasing the surface area available for infection and sporulation. Ceulemans *et al.* [34] compared the leaf area of: a *P. deltoides* clone, a *P. trichocarpa* clone, and of a *P. deltoides* x *P. trichocarpa* hybrid (*P. x generosa*). They found twice the leaf area index (LAI) for *P. x generosa* (LAI = 11.2) relative to the *P. deltoides* (LAI = 6.2) clone or the *P. trichocarpa* (LAI = 6.3) clones. If this pattern is consistent within the *P. balsamifera* x *P. deltoides* hybrid complex, larger LAI may correspond to greater infection frequency and increased inoculum production in hybrid stands. In order to best predict disease levels in natural stands and plantations of *Populus* species and their hybrids, a better understanding of these parameters is required.

In conclusion, the genetic characterization of the Red Deer River hybrid complex has indicated the presence of a small number of advanced generation hybrids not detected in previous studies. Furthermore, it has indicated that introgression is potentially related to the size of the local pollen cloud to which a particular female tree is exposed. In terms of disease resistance, the results from this study do not support the ‘hybrid susceptibility’ hypothesis. Rather, the pattern of susceptibility seen across the three genotype classes can best be explained by the ‘hybrid intermediacy hypothesis’ described by Fritz *et al.* [2]. The results also support the ‘phenological sink’ hypothesis, both in terms of the variability of hybrid genotypes compared to pure species and the variability in the bispecific B — D zone compared to the pure B and D zones. This implies that a variety of different genotypes with different dates of first leaf emergence may be more likely to ensure that, regardless of other epidemiological factors, there is susceptible tissue present early in the growing season resulting in pathogen establishment and potential epidemic development. However, it would be necessary to formally test this hypothesis by monitoring disease levels in mixed stands or plantations across different environments to determine the true impact of phenology on the epidemiology of this system.

## Supporting Information

Figure S1 **Key used to determine the date of first leaf emergence in the common garden experiment.** First leaf emergence has occurred in images A and C, whereas the first leaf was not considered emerged in images B and D.
(TIF)

Figure S2 **Key used to score date of bud set for the common garden experiment.** Images A and C are trees that are still actively growing, whereas bud set would have been considered to have occurred in images B and D.
(TIF)

Table S1 **Summary of Populations sampled along the Red Deer River Drainage in southern Alberta Canada including genotype class (B  =** ***P. balsamifera*** **, D  =** ***P. deltoides*** **, and H  =** ***P. deltoides*** **x** ***P. balsamifera*** **).**
(DOCX)

Table S2 **Provenance list of the individuals used as training populations for each** ***Populus balsamifera*** **and** ***P. deltoides*** **species with Structure version 2.3.3. All genotypes were from the Isabel** ***et al.*** **** [**22**] **study.**
(DOC)
