# Supplementary figures and images for: Testing the ‘Hybrid Susceptibility’ and ‘Phenological Sink’ Hypotheses Using the P. balsamifera – P. deltoides Hybrid Zone and Septoria Leaf Spot [Septoria musiva]
Source: PLoS One. 2013 Dec 27;8(12):e84437. doi: 10.1371/journal.pone.0084437 (PMC3874013; doi:10.1371/journal.pone.0084437)

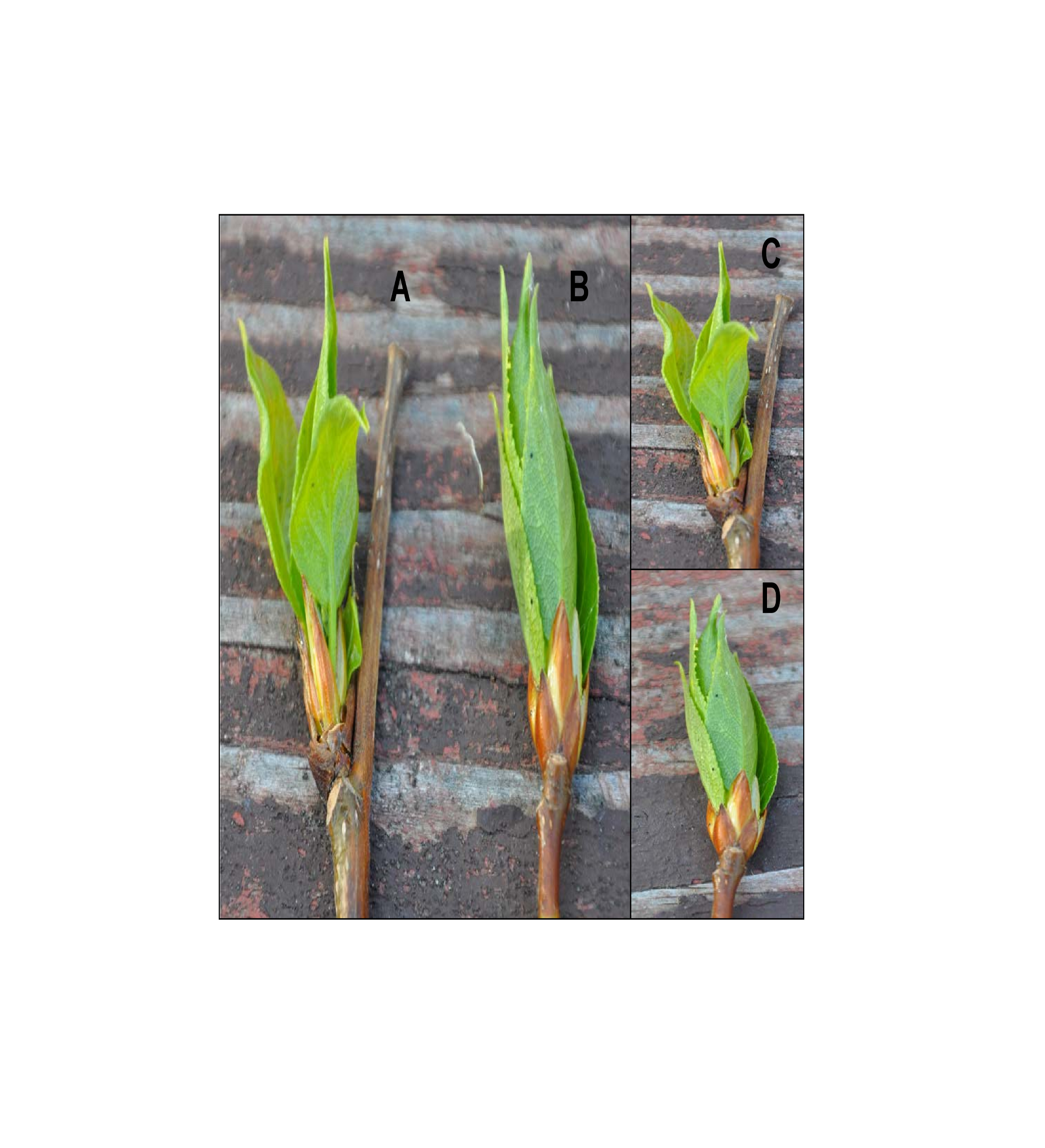

Supplement: Figure S1 — Key used to determine the date of first leaf emergence in the common garden experiment. First leaf emergence has occurred in images A and C, whereas the first leaf was not considered emerged in images B and D. (TIF) [file pone.0084437.s001.tif]

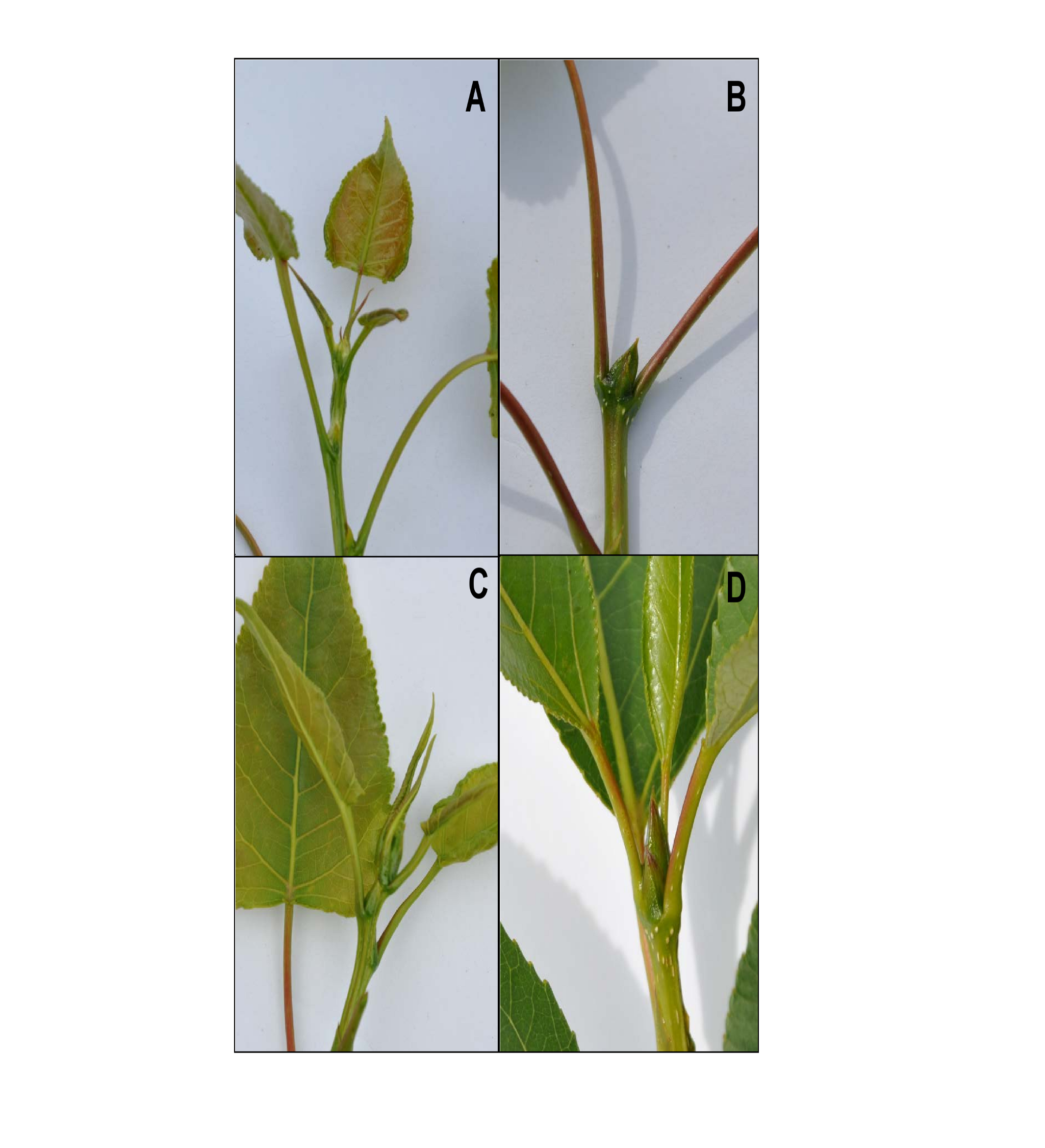

Supplement: Figure S2 — Key used to score date of bud set for the common garden experiment. Images A and C are trees that are still actively growing, whereas bud set would have been considered to have occurred in images B and D. (TIF) [file pone.0084437.s002.tif]
