# Supplementary material for: Testing the ‘Hybrid Susceptibility’ and ‘Phenological Sink’ Hypotheses Using the P. balsamifera – P. deltoides Hybrid Zone and Septoria Leaf Spot [Septoria musiva]
Source: PLoS One. 2013 Dec 27;8(12):e84437. doi: 10.1371/journal.pone.0084437 (PMC3874013; doi:10.1371/journal.pone.0084437)
Supplement: Table S1 — Summary of Populations sampled along the Red Deer River Drainage in southern Alberta Canada including genotype class (B = P. balsamifera , D = P. deltoides , and H = P. deltoides x P. balsamifera ). (DOCX) [file pone.0084437.s003.docx]

**Table S1. Summary of Populations sampled along the Red Deer River Drainage in southern Alberta Canada including genotype class (B = *P. balsamifera*, D = *P. deltoides*, and H = *P. deltoides* x *P. balsamifera*).**

| Population | Zone | No. of tree sampled | No. *P. balsamifera* | Lat. (N) | Long. (W) |
| --- | --- | --- | --- | --- | --- |
| PB | B | 1 | 1B, 0D, 0H^1^ | 53.60 | -115.00 |
| RV | B | 1 | 1B, 0D, 0H | 52.41 | -114.96 |
| J23 | B | 4 | 4B, 0D, 0H | 52.07 | -113.99 |
| J24 | B | 6 | 6B, 0D, 0H | 52.14 | -113.97 |
| J22 | B | 2 | 2B, 0D, 0H | 52.25 | -113.91 |
| J17 | B | 6 | 2B, 0D, 4H | 52.03 | -113.95 |
| J20 | B | 4 | 4B, 0D, 0H | 52.27 | -113.79 |
| JB | B | 19 | 19B, 0D, 0H | 52.27 | -113.59 |
| J21 | B | 4 | 4B, 0D, 0H | 52.33 | -113.91 |
| J19 | B | 4 | 3B, 0D, 1H | 52.24 | -113.50 |
| J18 | B | 3 | 3B, 0D, 0H | 52.31 | -113.08 |
| MX | H | 2 | 2B, 0D, 0H | 52.03 | -112.95 |
| J16 | H | 3 | 0B, 0D, 3H | 51.57 | -112.88 |
| SL | H | 21 | 0B, 5D, 16H | 51.65 | -112.90 |
| J14 | H | 3 | 0B, 3D, 0H | 51.46 | -112.75 |
| J15 | H | 6 | 2B, 2D, 2H | 51.46 | -112.76 |
| J13 | H | 2 | 0B, 0D, 2H | 51.42 | -112.63 |
| J12 | H | 1 | 0B, 0D, 1H | 51.40 | -112.59 |
| J11 | H | 2 | 0B, 2D, 0H | 51.28 | -112.33 |
| EB | H | 43 | 12B, 11D, 20H | 50.92 | -111.90 |
| J10 | D | 4 | 0B, 4D, 0H | 50.84 | -111.61 |
| DP | D | 1 | 0B, 1D, 0H | 50.76 | -111.52 |
| J9 | D | 1 | 0B, 1D, 0H | 50.78 | -111.32 |
| J8 | D | 1 | 0B, 1D, 0H | 50.74 | -111.27 |
| J7 | D | 1 | 0B, 1D, 0H | 50.74 | -111.22 |
| JENN | D | 19 | 0B, 19D, 0H | 50.84 | -111.15 |
| J6 | D | 3 | 0B, 3D, 0H | 50.92 | -111.06 |
| J5 | D | 1 | 0B, 1D, 0H | 50.91 | -110.84 |
| J4 | D | 2 | 0B, 2D, 0H | 50.85 | -110.69 |
| BL | D | 17 | 0B, 17D, 0H | 50.90 | -110.30 |
| J3 | D | 3 | 0B, 3D, 0H | 50.86 | -110.32 |
| J2 | D | 5 | 0B, 5D, 0H | 50.96 | -110.01 |
| J1 | D | 3 | 0B, 3D, 0H | 50.73 | -110.09 |
|  | Total | 205 | 65B, 85D, 49H |  |  |

^1^ Trees identified as F1 and backcrosses are in the same genotype classes.
