# Supplementary material for: Testing the ‘Hybrid Susceptibility’ and ‘Phenological Sink’ Hypotheses Using the P. balsamifera – P. deltoides Hybrid Zone and Septoria Leaf Spot [Septoria musiva]
Source: PLoS One. 2013 Dec 27;8(12):e84437. doi: 10.1371/journal.pone.0084437 (PMC3874013; doi:10.1371/journal.pone.0084437)
Supplement: Table S2 — Provenance list of the individuals used as training populations for each Populus balsamifera and P. deltoides species with Structure version 2.3.3. All genotypes were from the Isabel et al. [22] study. (DOC) [file pone.0084437.s004.doc]

**Table S2.** **Provenance list of the individuals used as training populations for each *Populus balsamifera* and *P. deltoides* species with Structure version 2.3.3. All genotypes were from the Isabel *et al.* [22] study.**

| Species | Country of origin | # individuals | Lat. (N) | Long. (W) |
| --- | --- | --- | --- | --- |
| *Populus balsamifera* | Canada | 1 | 50.22N | 63.63W |
|  |  | 1 | 48.22N | 67.18W |
|  |  | 2 | 46.40N | 67.25W |
|  |  | 2 | 47.57N | 68.65W |
|  |  | 2 | 48.92N | 68.74W |
|  |  | 2 | 58.02N | 68.65W |
|  |  | 2 | 45.82N | 73.10W |
|  |  | 2 | 42.98N | 81.25W |
|  |  | 1 | 50.08N | 91.90W |
|  |  | 1 | 53.20N | 99.38W |
|  |  | 2 | 50.37N | 101.70W |
|  |  | 2 | 56.27N | 104.23W |
|  |  | 2 | 59.23N | 105.72W |
|  |  | 1 | 56.92N | 111.50W |
|  |  | 1 | 54.75N | 118.63W |
|  | USA | 1 | 44.91N | 69.82W |
|  |  | 1 | 44.69N | 70.98W |
|  |  | 2 | 65.76N | 163.52W |
|  |  | 2 | 64.50N | 165.42W |
| *Populus deltoides* | Canada | 3 | 46.78N | 71.33W |
|  |  | 1 | 46.64N | 71.64W |
|  |  | 3 | 46.08N | 73.18W |
|  |  | 3 | 43.05N | 79.08W |
|  |  | 1 | 50.00N | 86.83W |
|  |  | 1 | 52.12N | 106.63W |
|  |  | 1 | 50.67N | 107.80W |
|  |  | 1 | 50.41N | 114.43W |
|  | USA | 1 | 38.48N | 85.48W |
|  |  | 1 | 33.00N | 88.13W |
|  |  | 1 | 35.12N | 89.92W |
|  |  | 1 | 35.48N | 90.00W |
|  |  | 1 | 34.36N | 90.59W |
|  |  | 2 | 33.70N | 90.96W |
|  |  | 1 | 32.85N | 91.12W |
|  |  | 1 | 35.40N | 97.60W |
|  |  | 7 | *unknown* | *unknown* |
